# Characterization of plant growth-promoting bacteria from *Vicia faba* root nodules grown on oasis soils and their potential to enhance soil fertility and crop growth attributes in arid regions

**DOI:** 10.1371/journal.pone.0353365

**Published:** 2026-07-14

**Authors:** Oumaima Chaieb, Souhir Abdelkrim, Iris Bertani, Wael Taamalli, Khediri Mannai, Fatma Souissi, Moez Jebara, Vittorio Venturi, Salwa Harzalli Jebara

**Affiliations:** 1 Laboratory of Legumes and Sustainable Agro-systems, Center of Biotechnology of Borj Cedria, Hammam Lif, Tunisia; 2 High Agronomic Institute of Chott Mariem, University of Sousse, Sousse, Tunisia; 3 Bacteriology Group, International Centre for Genetic Engineering and Biotechnology (ICGEB), Trieste, Italy; 4 Laboratory of Olive Biotechnology, Center of Biotechnology of Borj Cedria, Hammam Lif, Tunisia; 5 Higher Institute of Biotechnology of Beja, University of Jendouba, Beja, Tunisia; 6 African Genome Center, University Mohammed VI Polytechnic (UM6P, Ben Guerir, Morocco; Universidade de Coimbra, PORTUGAL

## Abstract

Soil degradation and nutrient depletion are major constraints in arid regions, particularly in fragile oasis ecosystems. Harnessing beneficial plant-bacteria interactions offers a sustainable approach to improving soil fertility and crop productivity. This study aimed to isolate and characterize plant growth-promoting rhizobacteria (PGPR) from root nodules of *V. faba* L. var. *minor* Saber 02 grown on 12 oasis soils, and to evaluate their potential for enhancing plant growth and soil fertility. Sixty bacteria were isolated from V. faba root-nodules. 16S rDNA sequencing revealed seven bacterial orders: *Enterobacteriales, Pseudomonodales*, *Burkholderiales*, *Xanthomonadales*, *Rhodospirillales*, *Hyphomicrobiales*, *Bacillales*. Plant growth promoting (PGP) traits screening identified five of the sixty isolates including Rhizobium laguerreae (Vf19), Bacillus halotolerans (Vf43), Gluconobacter frateurii (Vf47), Pseudomonas reinekei (Vf48), and Kosakonia radicincitans (Vf49) which harbor multiple PGP traits with interesting biocontrol potential as well as high tolerance to osmotic stresses. Eleven inoculums formed by mixing efficient and resistant PGPR to inoculate *V. faba* L. *minor* var. Saber 02 in contrasting soil fertility showed that co-inoculation with R. laguerreae Vf19 and B. halotolerans Vf43 significantly increased shoot biomass and nitrogen content. Likewise, inoculation with consortia formed by mixing R. laguerreae Vf19 + B. halotolerans Vf43 and R. laguerreae Vf19 + B. halotolerans Vf43 + K. radicincitans Vf49 improved soil total nitrogen levels by up to 2.5-fold in low-fertility soil. These findings highlight the potential of selected PGPR strains, particularly *R. laguerreae* Vf19, *B. halotolerans* Vf43, and *K. radicincitans* Vf49, as promising candidates for developing effective biofertilizers in management programs for sustainable agriculture in low fertility oasis soils to improve soil health, plant growth and productivity.

## Introduction

Climate change is currently regarded as one of the most important global environmental challenges, affecting natural ecosystems worldwide [1]. In drylands, extreme temperatures, water scarcity, soil salinity, wind erosion, soil mismanagement practices, low nutrient reserves have led to land degradation and desertification [2]. These soil issues pose serious threats to the sustainability of ecosystems especially to fragile ones like oasis. Nevertheless, oasis are among the most productive and vulnerable ecosystems in dryland, and have played a crucial role, since ancient times, in agricultural and the socio-economic development of these areas [1]. Further, oasis serve as important ecosystems supporting diverse habitats for flora and fauna [3].

Within the arid area, the cultivation of date palm (*Phoenix dactylifera* L.) as the main crop in oasis agroecosystems contributes to mitigating desertification and erosion and preserving the oasis microclimate, besides providing the main source of income for local farmers and the national economy [3,4]. Moreover, date palm microclimate favors agricultural production and plays an important role in the development of underlying crops and offsetting the effects of environmental stresses. However, to ensure good yields, many farmers increase the frequency of watering and intensive application of chemical fertilizers as well as excess agricultural practices (e.g., tillage) that dramatically affects soil properties, the quality of the irrigation water, and the diversity of the beneficial soil microbiota, important for plant and soil health [5]. This situation is worsened by intense climate changes, which impart severe impacts on plant–soil–microorganism interactions, thereby seriously threatening date palm growth and development and hampering the sustainability of these fragile ecosystems [4]. Therefore, the conservation and maintenance of date palm ecosystems and productivity are becoming increasingly challenging. To address these constraints, several sustainable strategies have been developed, including the integration of leguminous, which play a crucial role in maintaining ecosystem functions and soil quality.

In arid regions, leguminous plants are considered promising candidates for sustainable and ecological initiatives and programs to offset the negative effects of soil degradation and climate change [6]. Besides their ecological role in building soil fertility through their ability to form a symbiotic relationship with rhizobial species, legumes are also good colonizers of poor soils under extreme climatic conditions and thus contribute to preventing erosion [7]. Furthermore, the use of legumes in the cropping system not only increase nitrogen fixation but also improve crop production and soil fertility since they are “built-in” soil regulators through several other attributes [8–10]. Legume root-nodules are typical structures harbouring several bacterial species that coexist with rhizobia, present inside the nodules, are collectively known as non-rhizobial endophytes [11,12]. The bacteria lifestyle may directly or indirectly assist during the infection and colonization processes of the rhizobium-host association and are co-ordinately involved in the adaptation of host plants to their environment which is crucial for enhancing ecosystem resilience and complexity [13]. Therefore, the exploitation of the relationship between legumes and their root-nodules associated bacteria have been proposed as an alternative to improve the nitrogen input into the plant-soil system under various stressful conditions [14,15]. In this context, various genera of bacteria including *Pseudomonas*, *Bacillus*, *Rhizobium*, *Variovorax*, *Glucanobater*, *Azotobacter* and *Azospirillum* were recognized as plant growth-promoting rhizobacteria (PGPR) and have been reported to prevent the deleterious effects of environmental stresses and to promote the growth of host plant by several mechanisms such as production of phytohormones, solubilization of minerals, nitrogen fixation ability, siderophore production, and the suppression of phytopathogens for development of eco-friendly sustainable agriculture [16,17]. Moreover, certain strains possess the ability to produce biofilms by secreting extracellular polysaccharides under drought conditions, which helps in aggregating soil particles, thereby enhancing nutrient mobilization, particularly of iron and phosphate, and promoting plant growth in arid agroecosystems. Therefore, focusing on beneficial microorganisms from arid and desertic lands would serve as valuable and innovating biotechnological tools for restoring and enhancing agricultural activity in desertic areas in general and in oasis in particular [4].

Faba bean (*Vicia faba* L.) is one of the major cool season legume crops cultivated in semi-arid environment worldwide [18]. Under global warming and climatic change, *V. faba* is probably one of the best performing crops due to its ability to grow under all different climatic conditions as well as its high adaptability to a variety of soil environments [19]. Likewise, desert indigenous bacteria are increasingly recognized as a long-term environmental and ecological potential solution to sustain agriculture in the oasis ecosystem. Indeed, desert microorganisms are anticipated to play a key role in addressing critical agricultural challenges. However, there is a lack of literature that deals with bacteria of faba bean nodules when cultivated in oasis soils.

Recently, researchers have described soil fertility and the current situation in oasis as “a crisis” [1,20]. This issue has attracted considerable attention from governments and international organizations, underscoring the urgent need for sustainable management strategies to ensure ecological security and enhance the long-term resilience of these vulnerable ecosystems. Oasis sustainability can be met through the use of the association between bacteria native to arid lands and legumes adapted to harsh environments.

The main objectives of this research study were: (i) isolation and characterization of bacteria from the *Vicia faba* L. var. *minor* Saber 02 root-nodules grown in oasis soils; (ii) screening of bacteria strain for their PGP activities, symbiotic effectiveness and their tolerance to abiotic stresses; (iii) study the impact of *Vicia faba* L. var. *minor* Saber 02 -PGPRs interaction on oasis soil fertility and plant growth.

## Materials and methods

### Sampling and soil physico-chemical analysis

**Fig 1:**
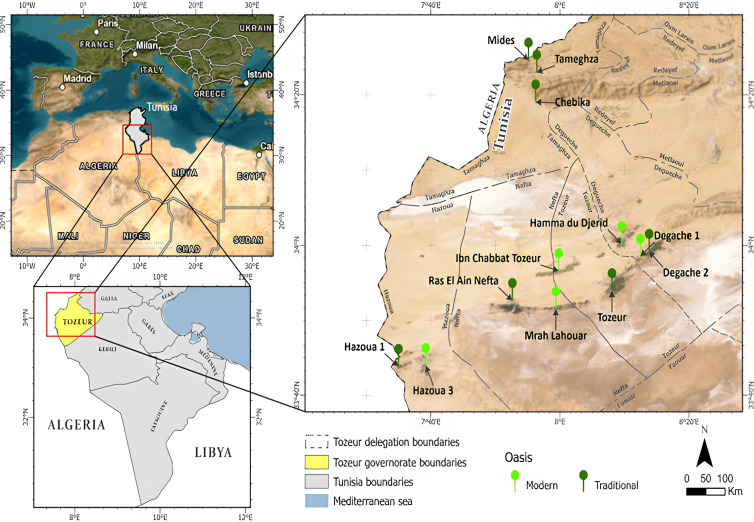
Geographical location and description of selected oasis [21–23].

The sampling was carried out from twelve oasis ecosystems (Fig 1) [21–23]. in the south-western part of Tunisia, in the Governorate of Tozeur, situated between two salt lakes (Chott El Djerid to the south and Chott El Gharsa). Sampling was authorized by the respective owners at each location. In each oasis, the soil samples were randomly collected from depth layers of 0–20 cm using an auger with three replications.

The collected soil samples were air-dried, sieved at 2 mm, and transferred to the laboratory. For the chemical analysis of soil samples, the electrical conductivity (EC) was analysed in a saturated soil paste; the soil pH was determined in (1:2.5) in water suspension, the percentage of organic matter (OM) was measured by thermo gravimetric analysis following calcination process [24]. Total nitrogen content (N) was determined by Kjeldahl method [25]. Soil available phosphorus (P_2_O_5_) in soil samples was measured using Olsen’s method [26] with the following modifications suggested by Dabin [27] by adding sodium bicarbonate and ammonium fluoride. Soils potassium content (K_2_O) was determined after extraction with ammonium acetate leaching as described by Mountier et al. [28].

### Plant cultivation and isolation of nodule-associated bacteria

Seeds of *Vicia faba* L. var. *minor* Saber 02 were surface sterilized with 70% ethanol for 1 min, washed several times with sterile water. Surface-sterilized seeds were germinated at 28 °C on moist filter paper kept in sterile Petri dishes. After 3 days, seedlings were planted into plastic pots containing the sampled soils and irrigated with a nitrogen-free nutrient solution, as described by Vadez et al. [29]. At flowering stage, plants were harvested. Shoots, roots and nodules were separated and the number of nodules was recorded. Plant organs were dried at 70 °C for 72 h; then shoots and roots dry weights were determined.

Collected nodules were washed with sterile water; their surfaces were sterilized by 70% ethanol and 0.2% HgCl_2_ and extensively washed with sterile distilled water according to the method described by Somasegaran and Hoben [30]. After that, nodules were crushed and the resulting suspension was streaked on the yeast extract mannitol agar (YEMA) plates [31] and incubated at 28°C for 3–6 days. Single colony were selected and isolated by repeated streaking on the YEMA medium to obtain pure culture. All isolates were preserved in glycerol 20% at −80 °C.

### Identification of selected isolates by 16S rRNA gene

DNA was extracted from each isolate using the method reported by Ahumada et al. [32]. PCR reaction was performed using the universal primers: fD1 (5’-AGAGTTTGATCCTGGCTCAG-3’) and rP2 (5’ ACGGCTACCTTGTTACGACTT-3’) as described by Weisburg *et* al. [33]. The reaction mixture contained 2.5 μM of each primer pair fD1 and rP2, 2.5 μl buffer 10× (20 mM Tris-HCl (pH 8.4), 50 mM KCl), 2.5 μl dNTPs, 1.5 μl of MgCl_2_ (25 mM), 0.20 μl Taq DNA polymerase, and approximately 100 ng genomic DNA as template. PCR cycle conditions were as follows: template DNA was denatured at 94°C for 4 min then the PCR was carried out for 35 cycles (1 min at 94°C, 1 min at 55°C, 2 min at 72°C, for each cycle). Finally, a 7 min extension period at 72°C was performed.

Isolates were identified by sequencing. 16S rDNA sequences were analysed with the nucleotide database available at National Centre for Biotechnology Information NCBI database (www.ncbi.nlm.nih.gov) using the basic local alignment search tool (BLAST) algorithm. Multiple sequence alignment and phylogenetic analysis was performed using Bioedit software and the 16S rRNA sequences were submitted in GenBank (NCBI).

### In vitro screening of bacterial strains for their plant growth promoting (PGP) activities

All isolated bacteria were studied for their PGP properties: production of indole acetic acid (IAA), siderophore, hydrocyanic acid (HCN), ammonia, 1-aminocyclopropane-1- carboxylate deaminase (ACCD), phosphate solubilization, N_2_ fixation and production of hydrolytic enzymes like cellulase, lipase and exopolysaccharide production.

### IAA production

Qualitative test for IAA production was carried out as described by Glickmann and Dessaux [34] with some modifications. Each bacterial strain was incubated in 10 mL of sucrose minimal salts (SMS) medium (g L^−1^: sucrose, 10; (NH_4_)_2_SO_4_, 1; K_2_HPO_4_, 2; MgSO_4_7H_2_O, 0.5; yeast extract, 0.5; CaCO_3_, 0.5; NaCl, 0.1; pH, 7.2) supplemented with 0.5 mg. mL^-1^ of tryptophane for 5 days at 28 °C on shaking condition at 150 rpm. Fully grown cultures were then centrifuged at 10000 rpm for 10 min and 1 mL of supernatant was mixed with 100 mL of ortho-phosphoric acid (10 mM) and 2 mL of Salkowsky reagent (2% 0.5 M FeCl_3_ in 35% perchloric acid) and incubated at 28°C in darkness for 30 min. The optical density (OD) was recorded at 530 nm. The level of IAA produced was estimated against the IAA standard.

### Phosphate solubilization

Bacterial strains were tested for their ability to solubilize tricalcium phosphate (Ca_3_(PO_4_)_2_) as described by Nautiyal [35] using National Botanical Research Institute’s phosphate (NBRIP) medium (g L^−1^: glucose, 10; Ca_3_(PO_4_)_2,_ 5; MgCl_2_ 6H_2_O, 5; MgSO_4_ 7H_2_O, 0.25; KCl, 0.2; (NH_4_)_2_SO_4_, 0.1; agar, 15; pH, 7). The NBRIP agar medium was inoculated with 20 µL of bacterial culture. The Petri plates were incubated at 28 °C for 7 days until the formation of transparent “halos” around each colony, due to the ability of bacteria to solubilize insoluble phosphate.

### Siderophores production

Siderophores production was detected by using the universal Chrome Azurol Sulphonate (CAS) assay as described by Schwyn and Neilands [36]. CAS agar plates was inoculated with bacterial cultures and incubated for 7 days at 28°C. The appearance of an orange halo around the colonies on blue agar was indicative of siderophore production.

### Hydrogen cyanide (HCN) production

The detection of cyanogenic bacteria was proved qualitatively by adapting the Feigl-Anger method [37]. Feigl-Anger paper was prepared by dipping filter paper in chloroform solution with copper ethylacetoacetate and tetra base (4, 4′ tertramethyldiaminodiphenylmethane) and left to air dry. Bacterial strains were streaked on LB agar plates; the paper was placed in the top of plates. The plates were sealed and incubated at 28°C for 7 days. The paper reacts with HCN gas and an oxidation product of the tetra base gives a blue colour recorded as weak (+), moderate (++), or strong (+++) reaction respectively.

### Cellulase production

Cellulase production was determined using the method of Kasana et al. [38]. Carboxymethyl cellulose (CMC) agar plates were inoculated with 5 𝜇l of overnight individual bacterial strains and incubated at 28°C for 72 h. The plates were flooded with 0.1% Congo red for 20 mn and then with 1 M NaCl for 20 mn. Positive indication of cellulase production is surrounded by clear halos zone around the colonies.

### Ammonia production

Estimation of ammonia production was carried out in peptone water. Actively growing bacterial cultures were inoculated in 10 ml peptone water and incubated for 48–72 h at 28 °C. Bacteria were harvested by centrifugation at 10000 rpm for 10 min and Nesseler’s reagent (0.5 ml) was added. Development of slight yellow to brownish colour was considered to be a positive test for ammonia production.

### ACC deaminase activity and nitrogen fixation ability

The 1-aminocyclopropane-1-carboxylic acid (ACC) deaminase activity was tested as described by Penrose and Glick [39]. Positive ACC activity was determined by comparing the growth of bacterial isolates on M9 supplemented with 30 µmol of ACC (as sole N source) to positive ((NH4)_2_SO_4_ as N-source) and negative controls (M9 without ACC) after 4 days incubation at 28°C.

### Exopolysaccharides (EPS) production

The production of exopolysaccharides (EPS) was determined by the analysis of mucoid growth in the agar medium. Briefly, bacterial culture was cross-streaked on a yeast malt agar (YM agar) plate and incubated for 48 h at 28°C. Colony growth was visually inspected, and the intensity of the mucoid growth was scored [40].

### Lipolytic activity

The presence of lipolytic and proteolytic activities was determined by streaking the bacterial isolates on 1/6 TSA (Tryptic Soy Agar) medium amended with 1% Glyceryl tributyrin and 2% of powder milk [41].

### Tolerance to abiotic stresses

The isolates were tested for their salt tolerance ability by spot inoculating the isolates on yeast extract mannitol agar (YEMA) plates containing different concentrations of NaCl (0.10, 0.20, 0.30,0.40,0.50,0.70,0.80,0.90 and 1M) incubated at 25 ± 2 °C for 5 days and observed for growth. Tolerance to acid (4, 5 and 6) and basic (8, 9 and 10) pH was assessed by adjusting the medium with concentrated HCl (12N) and NaOH (3M), respectively. The tolerance of bacteria to high temperature was carried out in standard YEMA medium plates incubated at 40, 45 °C for 5 days.

Tolerance to drought stress was evaluated by adding 30% of polyethylene glycol (PEG-6000) totryptic soy broth (TSB) broth medium at 30% [42].

### Nodulation test of *Vicia faba*

This test was performed in plastic pots containing autoclave-sterilized sand. Sixty rhizobial and non-rhizobial strains were used for the inoculation of *Vicia faba* L. var. *minor* Saber 02. Seeds were surface sterilized, germinated on 0.9% agar in Petri plates and incubated at 28°C for 72h. A single colony from each strain was grown in an Erlenmeyer flask of liquid YEM medium [31] at 28°C on a rotating incubator (150 rpm) for 48 h. Two days old, seedlings were transferred into plastic pots and inoculated with bacterial suspension of each strain. The control plants were treated with of uninoculated YEM. Plants were grown in a greenhouse at 25 °C/ 19 °C (day/night), a relative humidity of 60%, a 16 h light/8 h dark photoperiod and watered with nitrogen-free nutrient solution [29]. At flowering stage, plants were harvested and subjected to nodule counting and dry matter weighting.

### *Nod*C and *nif*H genes amplification

The *V. faba-* nodulating *Rhizobium* strains were screened for the presence of the *nodC* and *nif*H genes by performing selective amplification of these genes using primers *nif*HF (5’TACGGNAARGGSGGNATCGGCAA 3’) and *nif*Hi (5’ AGCATGTCYTCSAGYTCNTCCA 3’) for *nif*H gene and *nod*CF (5’ AYGTHGTYGAYGACGGTTC 3’) and *nod*CI (5’ CGYGACAGCCANTCKCTATTG 3’) for *nod*C gene. The selection of primer sets, the PCR reaction set up and thermal profiling conditions were performed following the methodology of Laguerre et al. [43]. 2 𝜇L of the PCR products were checked by electrophoresis in 1.5% agarose gel stained with Sybr Safe DNA Gel Stain (Invitrogen) and the sizes of the amplified fragments were determined by comparison with the 1Kb plus marker (Invitrogen). Presence of amplified bands was observed using UV-transilluminator and the gel was photographed.

The total nitrogen amount was determined by the Kjeldahl method [25]. The Symbiotic effectiveness (%) was calculated using the formula: S.E. (%) = (A / B)*100; Where, S.E. = symbiotic effectiveness, A = the amount of nitrogen in the plant isolate inoculated, B = the amount of nitrogen with nitrogen control [44].

### Potentialities of bacterial inocula

#### Growth condition and bacterial inoculation.

To identify the most effective inocula for improving the growth of *Vicia faba* L. var. minor Saber 02 and enhancing oasis soil fertility, selected bacterial strains including *R. laguerreae* (Vf19), *B. halotolerans* (Vf43), *G. frateurii* (Vf47), *P. reinekei* (Vf48), and *K. radicincitans* (Vf49) were chosen based on their symbiotic effectiveness, plant growth promoting attributes, and tolerance to abiotic stresses, including NaCl, PEG, and alkaline pH. Their performance was subsequently evaluated under controlled conditions using two soils with contrasting physicochemical properties: soil S4, characterized by high fertility and productivity due to elevated levels of organic matter, total nitrogen, P₂O₅, and K₂O, and soil S9, which exhibits low nutrient availability and high electrical conductivity, representing a more restrictive environment for plant growth. This experimental enables the evaluation of *Vicia faba*–PGPR interactions and the identification of the most effective symbiotic associations for enhancing soil fertility in both high- and low-fertility soils for sustainable agricultural. The experimental design was a 2 x 13 factorial, with soil type and inoculation treatment as fixed factors.

*Vicia faba* L. var. *minor* Saber 02 seeds sterilization, germination and inoculation was performed as described in previous section: Plant cultivation and isolation of nodule-associated bacteria. After germination, plant seedlings were transferred into plastic pots containing the collected soil samples (S4 and S9). Eleven bacterial consortia were formed by mixing the bacterial strains. Uninoculated plants were used as control. Ten replicates for each treatment were performed.

The experiment was conducted in a greenhouse at 25 °C/19 °C (day/night), relative humidity of 60%, 16 h light/8 h dark photoperiod.

Co-inoculation details are as follow:

Uninoculated

*Rhizobium laguerreae*

*R. laguerreae* + *Kosakonia radicincitans*

*R. laguerreae* + *Gluconobacter frateurii*

*R. laguerreae* + *Pseudomonas reinekei*

*R. laguerreae* + *Bacillus halotolerans*

*R. laguerreae* + *K. radicincitans* + *B. halotolerans*

*R. laguerreae* + *G. frateurii* + *B. halotolerans*

*R. laguerreae* + *K. radicincitans* + *P. reinekei*

*R. laguerreae* + *K. radicincitans* + *G. frateurii*

*R. laguerreae* + *K. radicincitans* + *G. frateurii* + *B. halotolerans*

*R. laguerreae* + *K. radicincitans* + *P. reinekei* + *B. halotolerans*

*R. laguerreae* + *K. radicincitans* + *G. frateurii* + *P. reinekei* + *B. halotolerans*

At flowering stage plant were harvested and subjected to dry matter weighting (shoot, root and nodules, separately) and shoot nitrogen content determination. The rhizospheric soils of *Vicia faba* L. var. *minor* Saber 02 plants were collected, air- dried, sieved and then stored in small flasks for soil total nitrogen analysis.

### Plant nitrogen content

Total nitrogen content in plants was determined using the Kjeldahl method [25]. A sample (25 mg) was digested with sulfuric acid (96%) in the presence of a catalyst mixture (CuSO_4_^+^/K2SO_4_^+^Se) at temperatures ranging from 100 to 350 °C until complete digestion was achieved. The released ammonia was collected in a boric acid (H₃BO₃) solution and quantified by titration with H2SO4 (N/100).

### Statistical analysis

Statistical analysis was determined using the R software v4.03. Phylogenetic analysis of the 16S rRNA gene sequences was conducted with molecular evolutionary genetics analysis (MEGA) software, v11. Phylogenetic tree was constructed by using neighbour-joining method.

Data for all response variables, shoot dry weight (SDW), root dry weight (RDW), nodule dry weight (NDW), shoot nitrogen content and soil total nitrogen, were subjected to a two-way analysis of variance (ANOVA). The experimental design was a 2 x 13 factorial, with soil type (soil 4 and soil 9) and inoculation treatment (uninoculated control; single inoculation with *R. laguerreae* (R); and eleven co-inoculation combinations incorporating *K. radicincitans* (K), *G. frateurii* (G), *P. reinekei* (P) and *B. halotolerans* (B)) as fixed factors. Before performing the ANOVA, the assumptions of normality and homogeneity of variance were assessed using Shapiro-Wilk and Levene’s tests (alpha = 0.05), respectively. SDW and RDW satisfied these assumptions and were analyzed using untransformed data. In contrast, NDW, shoot N content and soil total N showed departures from normality and were natural log transformed to satisfy parametric requirements. Given the presence of significant soil x treatment interactions for all measured variables (p < 0.001), treatment effects were interpreted via simple main effects. Specifically, one-way ANOVA for the inoculation factor was performed independently at each level of soil using the pooled residual mean square from the two-way ANOVA as the error term. To control the family-wise error rate across these simple effect analyses, statistical significance was accepted at a Bonferroni-adjusted alpha level of 0.025. Post-hoc pairwise comparisons were conducted within each soil type using Tukey’s Honestly Significant Difference (HSD) test applied to the estimated marginal means. statistical analyses were performed in R version 4.5 [45] using the packages *emmeans* [46], *multcomp* [47] and *car* [48]. Figures were produced using *ggplot2* [49]. and *patchwork* [50].

## Results

### Physico-chemical characteristics of oasis soils

The coordinates and characteristic of the studied oasis sites in Tunisia are depicted in Fig 1 and Table 1. The oasis soils were characterized by sand to sandy loam textures except for S4 soil, which was a sandy clay loam soil. Alkaline to slightly alkalines soil conditions were reflected in the pH values ranging between 7.22 and 8.75. The EC of soils varied from 1.21 to 6.79 ms cm^-1^; the highest EC values were observed in the S2, S6, S7, and S9 soils, while the least values were registered in S1, S3, S4, 8, 11 12 oasis soils. Results also demonstrated that S3, S4 and S5 soils were rich in OM, N, P_2_O_5_ and K_2_O when compared to the other studied soils. In contrast, S6, S7, and S9 oasis soils exhibited the lowest organic matter content, ranging from 0.62% to 2.09%. Notably, S2, S8, and S12 soils showed markedly low OM levels, with values not exceeding 0.84%. In contrast, soil samples from S3, S4, and S5 demonstrated significantly higher organic matter levels, with the maximum value recorded at 4.57% in S5 oasis soil.

**Table 1:** Physico-chemical properties of prospected soils.

Soils	Soil texture (%)			OM (%)	Total N (%)	P_2_O_5_ (ppm)	K_2_O (ppm)	pH	EC (ms cm^-1^)
	Clay	Limon	Sand						
S1	9.32	3.98	86.71	2.94d	0.10de	111.94e	161.00b	7.22f	1.24i
S2	6.21	2.24	91.43	0.62h	0.03f	63.03g	76.52e	8.30bc	5.03c
S3	8.72	15.40	75.80	3.62c	0.19b	368.17b	162.03b	7.76e	1.63h
S4	22.800	2.70	74.51	4.09b B0b	0.23a	498.70a	218.21a	8.18 cd	2.78e
S5	10.60	10.17	79.22	4.57a	0.25a	333.94c	165.06b	8.05d	2.53f
S6	5.48	6.51	88.07	1.25g	0.09de	46.10h	79.17e	8.75a	6.80a
S7	5.17	8.77	85.32	2.09e	0.12 cd	88.20f	95.68d	8.20 cd	3.76d
S8	9.90	12.04	78.05	0.80h	0.11cde	135.03d	80.11e	7.68e	1.51h
S9	9.19	1.41	89.41	1.65f	0.09de	66.67g	60.12f	8.43b	5.85b
S10	8.37	10.20	81.44	2.07e	0.14c	120.00de	91.32d	8.11 cd	2.86e
S11	5.15	2.85	89.43	2.11e	0.07ed	89.75f	130.15c	8.02d	2.26g
S12	6.29	17.20	76.06	0.84h	0.04f	69.00g	59.58f	7.59e	1.21i

Values are means of three replicates, means followed by a common lowercase letter are not significantly different at *p* < 0.05, according to Tukey’s HSD test. OM: Organic Matter; Total N: Total Nitrogen; P_2_O_5_: available Phosphorus; K_2_O: Soil Potassium; EC: Electrical Conductivity.

Nitrogen content oscillated between 0.04% and 0.25%, with the highest amounts detected in S3, S4 and S5 soils. Furthermore, S4 soils exhibited the greatest level of available phosphorus and potassium concentration reaching 498.70 ppm and 218.21 ppm, respectively.

### Growth attributes of *Vicia faba* grown on oasis soils

**Fig 2:**
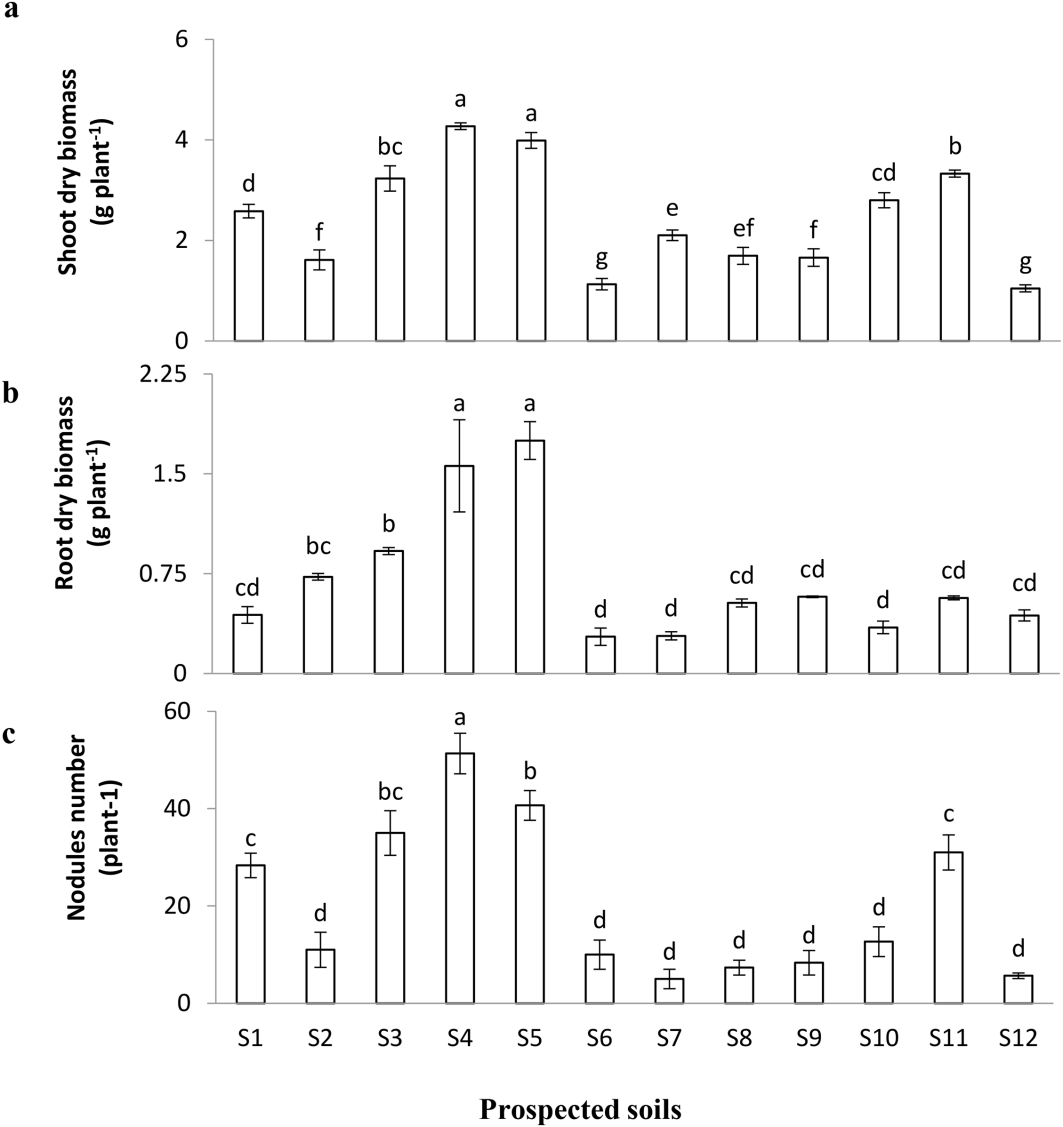
Growth parameters of *Vicia faba* L. var. *minor* Saber 02 grown in collected soil samples. **Plants were harvested at flowering stage.** a: Shoot dry biomass; b: Root dry biomass; c: Nodules number. Data presented as means (±SD) of five replicates. Bars with different letters are significantly different according to Tukey test, *p* < 0.05.

The best crop production was registered in S3, S4 and S5 oasis soils (Fig 2). Particularly, S4 and S5 soils appeared to be the most suitable for plant growth with the highest values of shoot dry weight (4.27 and 3.99 g plant^-1^, respectively), root dry weight (1.56 and 1.75 g plant^-1^, respectively), and nodule number (51 and 41 plant^-1^, respectively), compared to plants grown on the other soil samples. Conjointly, the second-highest nodules number was recorded in plants grown on S1, S3 and S11 soils (35; 31; 28, respectively). The lowest nodule number was found in plants grown on S7 soil, with an average of five small white nodules.

Surface sterilized root nodules obtained from this experiment allowed the isolation of 60 bacterial strains.

### 16S rDNA profiling

**Fig 3:**
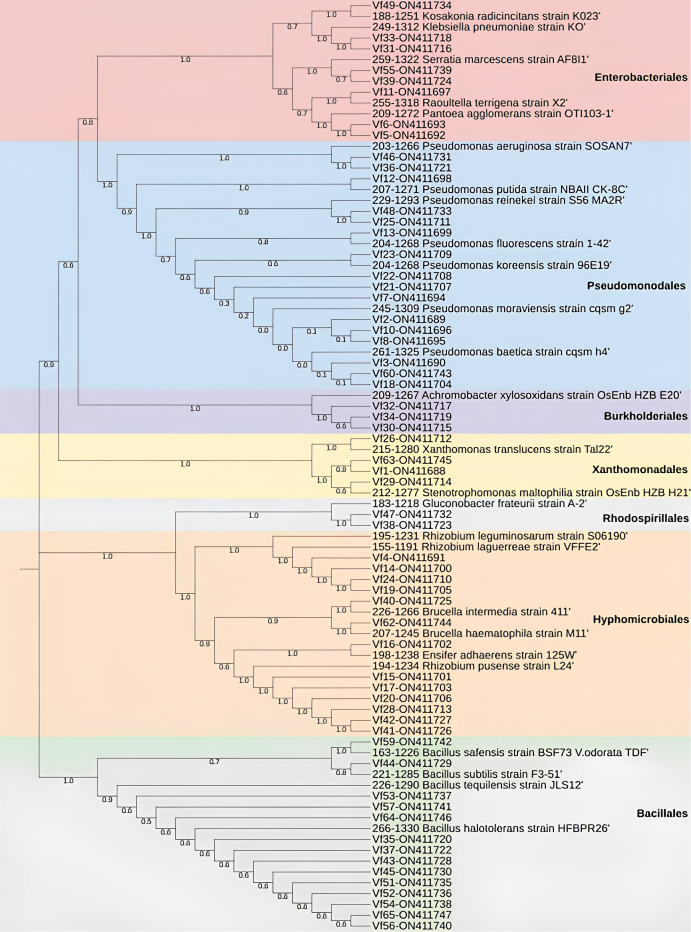
Neighbor-joining phylogenetic tree based on the partial sequence of the 16S rRNA gene of bacterial isolates from *Vicia faba* L. var. *minor* Saber 02 roots and their related type strains. The evolutionary distances were computed using Kimura 2-parameter model. Bootstrap values are given at branch nodes and are based on 1000 replicates (values higher than 50% are indicated). The strains were cited with their corresponding accession numbers. Bacterial strains in the same-colored box belong to the same order.

The 16S rRNA gene, of the sixty bacteria isolated from *Vicia faba* L. var. *minor* Saber 02 root nodules, was sequenced and a phylogenetic tree based on the 16S rRNA genes was obtained using the Neighbour-joining method (Fig 3). Seven major lineages, corresponding to seven bacterial orders (*Enterobacteriales, Pseudomonodales*, *Burkholderiales*, *Xanthomonadales*, *Rhodospirillales*, *Hyphomicrobiales*, *Bacillales*) and supported by the bootstrap analysis, were distinguished. A dendrogram generated from 16S rRNA data showed that the majority of strains (16 strains) belonged to the *Pseudomonodales* order followed by *Bacillales* (14 strains), *Hyphomicrobiales* (13 strains) and *Enterobacteriales* orders. However, only four, three, and two strains were grouped into *Xanthomonadales*, *Burkholderiales* and *Rhodospirillales* orders, respectively.

### PGP traits of *Vicia faba* symbiotic bacteria

Results revealed that 52/60 strains were found positive for more than two in vitro PGP traits which include solubilization of phosphate, production of IAA, siderophore, cellulose and ammonia (S1 Table).

### IAA production

The quantitative estimation of IAA production using tryptophan as a precursor revealed that all the strains were able to produce this phytohormone with varying levels ranging from 0.98 to 70.71 μg ml^-1^. *K. Radicincitans* Vf49 gave maximum auxin production followed by *B. haematophila* Vf62 and *S. maltophilia* Vf1 producing respectively 70.71 μg ml^-1^, 62.97 μg ml^-1^ and 53.18 μg ml^-1^. Among the rhizobial strains Vf19, Vf28, and Vf42 produced the highest amount of IAA.

### Phosphate solubilisation

As listed in S1 Table, fourteen bacteria exhibited a phosphate solubilisation capacity. Maximum activity was recorded in *G. frateurii* strains Vf47 and Vf38 (25.01 mm and 17.67 mm, respectively) followed by *S. marcescens* Vf55 (10.60 mm) and *K. radicincitans*Vf49 (8.17 mm). Phosphate solubilisation ability was not detected in rhizobial strains.

### Siderophore production

The ability of bacterial strains to produce and secrete iron-chelating compounds was detected in 96% of isolates as detected via orange halos around the colonies on CAS agar plates. As shown in S1 Table, the *Pseudomeunas* species Vf48 and Vf46 and *B. halotolerans* Vf45 exhibited a significant siderophore production (49.00 mm; 47.50 mm; 48.84 mm, respectively) followed by *B. intermedia* Vf40, *P. moraviensis*Vf23, *B. halotolerans* Vf54 and *K.radicincitans* Vf49. Among the rhizobial species Vf42, Vf24 and Vf19 have shown significantly siderophore production ability.

### Hydrogen cyanide (HCN) production

Among the sixty bacterial isolates, twenty-two were able to produce HCN (S1 Table). A strong HCN production capacity was recorded in all strains identified as *P. reinekei* while was restricted to the strains Vf3 and Vf48 of *P. fluorescens*. *G. frateurii* Vf47 and *S. marcescens* Vf55 showed a moderate HCN production, whereas *Rhizobium* strains Vf4, Vf17, Vf19 and Vf28 and *Bacillus* strains Vf51, Vf52, Vf53 and Vf54 produced low levels of HCN.

### Cellulase production

Twenty-four bacterial isolates were able to produce cellulase by forming a halo zone around bacterial colonies on CMC agar medium and the genera *Bacillus* presented 54% of the producers. Highest cellulose activity was recorded by *B. halotolerans* strains Vf35, Vf43, Vf51, Vf52 and Vf54. The *Rhizobium* strains Vf4, Vf14 and Vf19 also showed capacity to synthesize cellulase (S1 Table).

### Ammonia production

Results revealed that 75% of the bacterial strains demonstrated the capacity to produce ammonia. Likewise, all strains belonged to the *Pseudomeunas* genera were ammonia producers. Nonetheless, the highest amount was produced by *G. frateurii* Vf47 and *B. halotolerans* strains Vf35, Vf52 Vf54 and Vf65 (S1 Table). Obtained results also indicated that all strains belonged to the *Rhizobium* genera were able to synthesize ammonia except for strains Vf4 and Vf15.

### ACC deaminase activity

A strong AAC deaminase ability was registered by *R. laguerreae* strain Vf19, *K. radicincitans* Vf49 and *Achromobacter xyloxidans* Vf34 (S1Table). Further, among rhizobial and non-rhizobial strains 78% were able to grow in M9 complete medium.

### Exopolysaccharides (EPS) production

The production of exopolysaccharides (EPS) by bacterial isolates was evaluated by the inspection of mucoid growth of bacterial colonies in the YM agar medium. More than 80% of bacterial strains were able to produce EPS and rhizobial strains exhibited the highest potential. *K. radicincitans* Vf49 showed moderate EPS production ability (S1Table).

### Lipolytic activity

*B. halotolerans* strains Vf35, Vf43, Vf52 and Vf54, *S. maltophilia* species and some strains belonged to the *Pseudomeunas* genera (Vf8 and Vf36) were displayed significant lipolytic activity whereas it was not detected in rhizobial strains.

### Tolerance of bacterial strains to abiotic stresses

Results indicated that 73% of the bacterial strains were able to grow at NaCl concentrations up to 1 M (S2 Table). Strains belonging to the *Pseudomonas* and *Bacillus* genera exhibited the highest tolerance to NaCl, followed by *K. radicincitans* Vf49. *Rhizobium* strains demonstrated the ability to adapt to a broad range of salinity conditions, from 0.80 M to 1 M NaCl. In contrast, strains of *Raoultella terrigena* and *Pantoea agglomerans* were identified as the least tolerant.

The bacterial strains showed their ability to grow in a wide pH range and to survive in both acidic and alkaline mediums. Within the bacterial collection, all the isolates were able to grow in pH 10, while 52% of them were able to grow in pH 4 (S2 Table). *B. safensis* Vf59 and *B. subtilis* Vf44 were the least tolerant to acidic medium.

Most of the strains (93%) exhibited remarkable tolerance to high temperature (45°C). Whereas, *S. maltophilia* strains and the strain *X. translucens* Vf26 were the least tolerant and were able to grow at a temperature not exceeding 40 ºC*.*

The tolerance of strains to drought stress revealed that expect the strains *S. marcescens* Vf39, *X. translucens* Vf26 and *A. xylosoxians* Vf34 as well as *S. maltophilia* strains, all the other bacteria were able to grow in presence of 30% of PEG.

### Symbiotic effectiveness assay

Result showed that out of the 60 isolated strains, only four were capable of nodulating *Vicia faba* L. var. *minor* Saber 02: Vf4 and Vf14, identified as *R. leguminosarum*, and Vf19 and Vf24, identified as *Rhizobium laguerreae*. Moreover, results revealed that *R. laguerreae* strain Vf19 presented the highest number of nodules (49 nodules plant^−1^). As expected, no nodule formation was observed in control plants and plants inoculated with the other bacterial strains.

The potential of growth promotion of the isolated bacterial strains was assayed and found that 88% of the strains promoted dry weights of shoot (SDW) and root (RDW) parts (Table 2). Interestingly, a greater dry biomass increase was detected in shoots and roots by 3 and 5-fold, respectively, in plants inoculated with *Rhizobium laguerreae* Vf19 and *Kosakonia radicincitans*Vf49.

**Table 2:** Greenhouse evaluation of sixty bacterial strains isolated from *Vicia faba* L. var. *minor* Saber 02 root nodules for their effects on nodulation and plant growth promotion.

Accession NCBI Number	Bacterial strain	SDW	RDW	NN	Accession NCBI Number	Bacterial strain	SDW	RDW	NN
ON411688	Vf1	1.08g	0.77j	0	ON411718	Vf33	1.61cde	1.61def	0
ON411689	Vf2	1.08g	1.33h	0	ON411719	Vf34	0.73kl	1.30h	0
ON411690	Vf3	0.82ijk	1.39efgh	0	ON411720	Vf35	2.11b	2.05d	0
ON411691	Vf4	1.69c	1.78d	14,80c	ON411721	Vf36	1.11g	1.03i	0
ON411692	Vf5	0.84hij	0.68j	0	ON411722	Vf37	1.072gh	1.74d	0
ON411693	Vf6	0.75k	0.728j	0	ON411723	Vf38	1.09g	1.68d	0
ON411694	Vf7	1.40def	1.37gh	0	ON411724	Vf39	1.06gh	1.68d	0
ON411695	Vf8	1.07gh	1.31h	0	ON411725	Vf40	1.07gh	1.40efgh	0
ON411696	Vf10	1.63 cd	1.28h	0	ON411726	Vf41	1.20g	1.75d	0
ON411697	Vf11	1.48cde	1.76d	0	ON411727	Vf42	1.39ef	1.76d	0
ON411698	Vf12	0.77k	1.34h	0	ON411728	Vf43	1.61cde	2.10c	0
ON411699	Vf13	1.50cde	1.40efgh	0	ON411729	Vf44	0.51lmn	1.29h	0
ON411700	Vf14	1.46de	2.08c	16,80c	ON411730	Vf45	0.48mn	1.28h	0
ON411701	Vf15	0.75k	1.76d	0	ON411731	Vf46	0.71klm	1.29h	0
ON411702	Vf16	1.7c	1.80d	0	ON411732	Vf47	1.70c	1.77d	0
ON411703	Vf17	0.75k	1.01i	0	ON411733	Vf48	1.01ghij	1.80d	0
ON411704	Vf18	1.09g	1.32h	0	ON411734	Vf49	2.44a	2.49a	0
ON411705	Vf19	2.39a	2.36ab	49,20a	ON411735	Vf51	1.45de	1.29h	0
ON411706	Vf20	0.74g	1.02i	0	ON411736	Vf52	2.09b	1.0i	0
ON411707	Vf21	1.18g	1.35h	0	ON411737	Vf53	1.21gh	1.04i	0
ON411708	Vf22	0.79jk	1.40efgh	0	ON411738	Vf54	1.11g	1.66d	0
ON411709	Vf23	1.58cde	1.62de	0	ON411739	Vf55	0.80jk	1.38fgh	0
ON411710	Vf24	2.13b	2.17bc	28,20b	ON411740	Vf56	1.62 cd	1.35h	0
ON411711	Vf25	0.64klmn	1.29h	0	ON411741	Vf57	1.58cde	0.82ij	0
ON411712	Vf26	0.41n	0.60 j	0	ON411742	Vf59	1.10g	0.77j	0
ON411713	Vf28	1.20g	2.07c	0	ON411743	Vf60	2.30a	1.42efgh	0
ON411714	Vf29	1.10g	1.67d	0	ON411744	Vf62	1.62cde	1.27h	0
ON411715	Vf30	1.06gh	1.30h	0	ON411745	Vf63	1.08g	1.32h	0
ON411716	Vf31	2.13b	1.73c	0	ON411746	Vf64	0.46n	1.59defg	0
ON411717	Vf32	0.74kl	1.81d	0	ON411747	Vf65	1.05ghi	1.02i	0
					Un-inoculated	T	0.47n	0.78j	0

SDW: shoot dry weight, g plant^-1^; RDW: root dry weight, g plant^-1^; NN: nodules number, plant^-1^. Mean values followed by different letters indicate average of five replicates. Significant differences between inoculated and un-inoculated plants at the level p < 0.05 according to Tukey test.

In addition, noteworthy values of RDW were registered in plants inoculated with *R. laguerreae* Vf24, *Bacillus halotolerans* strains Vf35 and Vf52 and the strain *Pseudomonas fluorescens*Vf60. Concerning SDW, plants inoculated with the *Rhizobium* species Vf14, Vf24 and Vf28, and the strain *B. halotolerans* Vf43 exhibited a significantly higher increase compared to all inoculated plants (Table 2).

### *Nod*C and *nif*H genes amplification

Only four bacterial strains (Vf4, Vf14, Vf19 and Vf24) were selected based on their ability to nodulate *V. faba* L. *minor* var. Saber 02, for further screening the presence of the *nodC* and *nifH* gene. PCR amplification of *nod*C and *nif*H gene showed that the *nif*H gene was amplified for all the *V. faba-* nodulating *Rhizobium* strains (Table 3). The product of predicted size of 360–400 bp was obtained indicating the presence of nitrogen fixing genes, likewise the *nod*C gene was successfully amplified in *R. leguminosarum* and *R. laguerreae* strains.

**Table 3:** *Nod*C and *nif*H genes amplification and N_2_ fixation of *V. faba* L. *minor* var. Saber 02*-* nodulating *Rhizobium* strains.

Strain	Accession number	Closet relative species	Similarity (%)	Nod C	Ni*f* H	N_2_ fixation (%)
V*f* 4	ON411691	*Rhizobium leguminosarum*	99.90%	+	+	S (45%)
V*f* 14	ON411700	*Rhizobium leguminosarum*	99.81%	+	+	S (38%)
V*f* 19	ON411705	*Rhizobium laguerreae*	99.90%	+	+	S (100%)
V*f* 24	ON411710	*Rhizobium laguerreae*	99.81%	+	+	S (84%)

(+) Positive result, (-) negative result, S significant increase in N_2_ fixation comparing to the non-inoculated control as indicated between parentheses.

### Impact of PGPR inocula on *Vicia faba g*rowth and soil fertility in two contrasting soils

The bacterial strains used in this study were selected on the basis of their efficiency, plant growth promoting traits studied above, thus, from 60 strains, 11 inoculums were formed by mixing efficient and resistant PGPR (Table 4). In order to determine the symbiotic effectiveness of these inoculums and their applicability in the field and to demonstrate best symbiosis *V. faba*-PGPR in soil fertility enhancement for sustainable agriculture, all these inoculums were tested in the two selected soil S4 which demonstrate its best productivity due to richness in OM, total N, P_2_O_5_, K_2_O and S9 soil characterized low fertility levels and high EC.

**Table 4:** Plant growth-promoting traits and tolerance to abiotic stresses of selected bacterial strains isolated from *Vicia faba* L. var. *minor* Saber 02 root nodules.

Accession number	Species	IAA	Sid	PS	C	A	HCN	N_2_	ACC deaminase	EPS	LA	PEG 30%	Max NaCl (mM)
**ON411705**	***R. laguerreae* (*Vf*19)**	25.36c	23.51d	nd	+	++	+	+++	+++	+++	–	++	900
**ON411728**	***B. halotolerans* (*Vf4*3)**	39.62b	31.00c	nd	+++	+	–	++	+	–	+++	+++	1000
**ON411732**	***G. frateurii* (*Vf4*7)**	12.73d	12.00e	25.01a	–	+++	++	++	+	+	–	+	900
**ON411733**	***P. reinekei* (*Vf4*8)**	13.86d	49.00a	nd	–	+++	+++	+	–	+	–	+++	1000
**ON411734**	***K. radicincitans* (*Vf4*9)**	70.71a	35.00b	8.17b	–	–	–	+++	+++	++	–	++	1000

IAA: indoleacetic acid production, S: siderophore production, PS: phosphate solubilization capacity, C: cellulase activity, A: ammonia production, HCN: Hydrogen cyanide production, N_2_: Nitrogen fixation, ACC deaminase activity, EPS: exopolysaccharide production, LA: Lipolytic activity, PEG 30%: tolerance to drought stress, Max NaCl (mM): maximum NaCl concentration tolerated by bacterial strains. Values are means of three replicates. Intensity of production indicator: none, − ; weak, + ; moderate, ++; strong, +++. Means followed by a common lowercase letter are not significantly different at *p* < 0.05, according to Tukey’s HSD test, nd, not detected activities.

The two-way ANOVA demonstrated that plant growth parameters and soil nitrogen content were significantly influenced by the main effects of soil type and inoculation treatment, as well as their interaction (p < 0.001 for all variables), as detailed in Table 5. Given the significant soil x treatment interaction, treatment effects were interpreted separately within each soil type.

**Table 5:** Two-way ANOVA F-statistics and significance levels for the effects of soil type, inoculation treatment and their interaction on *Vicia faba* growth parameters and soil total nitrogen.

Variable	Source	df	F	p
**SDW (g plant**^**-1**^)	Soil	1	324.58	< 0.001
	Treatment	12	50.88	< 0.001
	Soil x Treatment	12	14.48	< 0.001
**RDW (g plant**^**-1**^)	Soil	1	11.38	< 0.001
	Treatment	12	28.24	< 0.001
	Soil x Treatment	12	10.62	< 0.001
**NDW (mg plant**^**-1**^)	Soil	1	15.64	< 0.001
	Treatment	12	114.76	< 0.001
	Soil x Treatment	12	24.45	< 0.001
**Shoot N content (%)**	Soil	1	126.02	< 0.001
	Treatment	12	29.9	< 0.001
	Soil x Treatment	12	4.81	< 0.001
**Soil total N (g kg**^**-1**^)	Soil	1	1963.7	< 0.001
	Treatment	12	40.3	< 0.001
	Soil x Treatment	12	13.49	< 0.001

Degrees of freedom (df), F-statistics and associated p-values are reported for each source of variation across all response variables. SDW: shoot dry weight; RDW: root dry weight; NDW: nodule dry weight. NDW, shoot nitrogen content and soil total nitrogen were log-transformed prior to analysis to satisfy parametric assumptions.

### Effect of PGPRs co-inoculation on *Vicia faba* growth attributes

**Fig 4:**
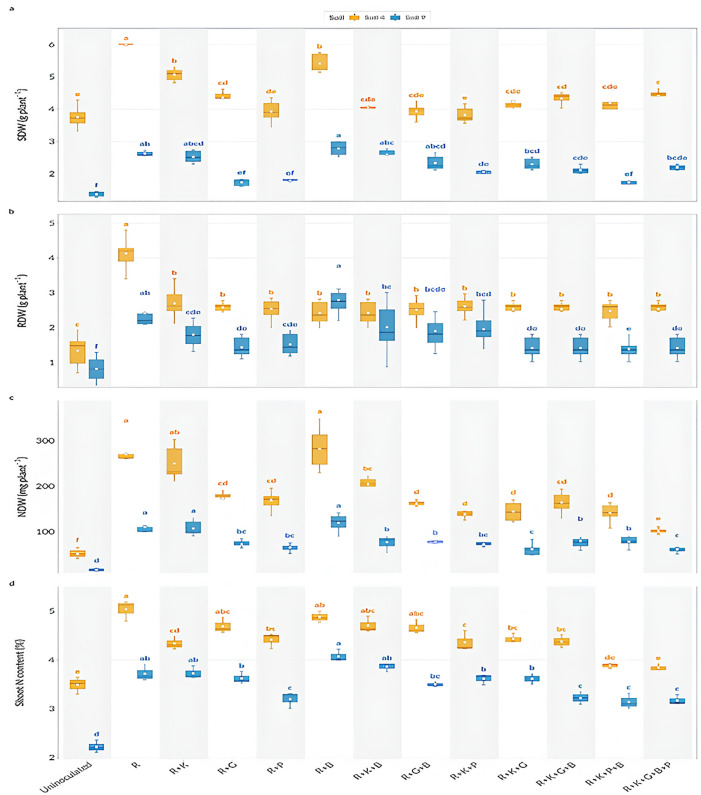
Effect of PGPR inoculation treatments on *Vicia faba* L. var. *minor* Saber 02 growth parameters at flowering stage in two contrasting soils (Soil 4 and Soil 9). Shoot dry biomass; b: Root dry biomass; c: Nodules dry biomass; d: Shoot nitrogen content. Box plots display the median (central line), interquartile range (box) and data spread (whiskers); the white diamond (◇) indicates the group mean. Given the significant soil × treatment interaction detected by two-way ANOVA (p < 0.001 for all variables), treatment effects were assessed via simple main effects analysis performed independently within each soil type, using the pooled residual mean square from the two-way ANOVA as the error term. Letters above boxes indicate Tukey HSD groupings from simple main effects contrasts performed independently within each soil type (alpha = 0.05). Treatments sharing the same letter within a soil type do not differ significantly. R: *Rhizobium laguerreae*; K: *Kosakonia radicincitans*; G: *Gluconobacter frateurii*; P: *Pseudomonas reinekei*; B: *Bacillus halotolerans*.

The efficacy of the bacterial inoculation treatments on *Vicia faba* L. var. *minor* Saber 02 growth attributes was highly dependent on the soil type (Fig 4). In soil 9, the dual inoculation of *R. laguerreae* + *B. halotolerans* (R + B) resulted in substantial increases in shoot dry weight (SDW; + 103%), root dry weight (RDW; + 241%), nodule dry weight (NDW; + 646%) and shoot nitrogen content (+84%) compared to the uninoculated control. While the R + B treatment achieved the highest numerical values across all growth parameters, it remained statistically comparable to the single inoculation with *R. laguerreae* and the dual inaoculation with *R.* laguerreae + *K. radicincitans* (R + K). In contrast, the performance hierarchy shifted in soil 4, where the single inoculation with *R. laguerreae* emerged as the most effective strategy. This treatment significantly enhanced SDW, RDW and NDW by 1.5-, 3- and 5.5-fold, respectively, compared to the control, while simultaneously maximizing shoot N content (1.5-fold increase).

### Effect of PGPRs co-inoculations on soil nitrogen content

**Fig 5:**
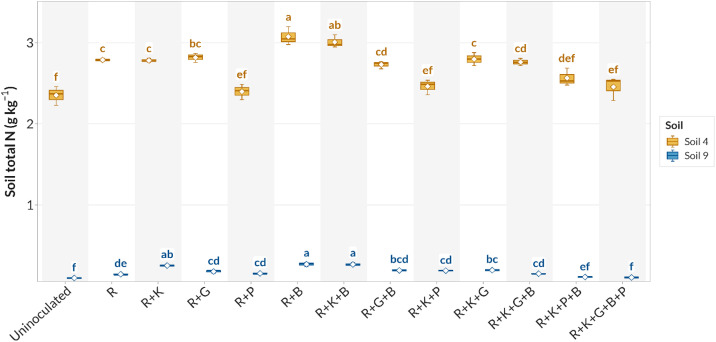
Effect of PGPR inoculation treatments on soil total nitrogen content in two contrasting soils (Soil 4 and Soil 9). Box plots display the median (central line), interquartile range (box) and data spread (whiskers); the white diamond (◇) indicates the group mean. Given the significant soil × treatment interaction detected by two-way ANOVA (p < 0.001), treatment effects were assessed via simple main effects analysis performed independently within each soil type, using the pooled residual mean square from the two-way ANOVA as the error term. Letters above boxes indicate Tukey HSD groupings from simple main effects contrasts performed independently within each soil type (alpha: 0.05). Treatments sharing the same letter within a soil do not differ significantly. R: *Rhizobium laguerreae*; K: *Kosakonia radicincitans*; G: *Gluconobacter frateurii*; P: *Pseudomonas reinekei*; B: *Bacillus halotolerans*.

Soil total nitrogen content followed distinct patterns based on bacterial inoculation and the soil type (Fig 5). In soil 9, the most pronounced enrichment was observed in the R + B, R + K + B and R + K treatments, where N levels were up to 2 times higher than those of the uninoculated control. Notably, bacterial inoculation with the four-strain (R + K + P + B) and five-strain (R + K + G + B + P) combinations failed to produce significant N increases compared to the control. Regarding soil 4, the R + B treatment again proved to be the most effective, resulting in the highest soil total N, with a 31% increase compared to the control, followed closely by the R + K + B consortium (28%). In contrast, treatments involving *P. reinekei* (R + P, R + K + P, R + K + P + B, and R + K + G + B + P) showed no significant difference from the uninoculated soil.

## Discussion

The assessment of soil fertility status in oasis ecosystems revealed that the majority of soils were slightly to moderately saline, with EC values ranging from 2.26 to 6.79 mS cm^-¹^. These differences can be due to the quality of the irrigation water and to the differences in gypsum content between soils or/and insufficient drainage in some oasis [51–53]. Soil pH ranged from alkaline to slightly alkaline (7.21–8.75), consistent with previous studies conducted in comparable soils in Tunisia [54–57]. Elevated pH levels could be attributed to the high salt contents, resulting from irrigation with water from saline aquifers and rises of the underground water table [51, 58].

Results also demonstrated that soil samples S3, S4 and S5 from the traditional oasis, exhibited significantly higher levels of OM, total N, P_2_O_5_, and K_2_O compared to the other soils studied. Indeed, traditional oasis systems depend on a symbiosis between animal husbandry and tillage, as animals provide manure for maintaining or restoring soil fertility [59]. Thus, the elevated OM levels in S3, S4 and S5 soils can be attributed to the regular application of organic amendments, the integration of suitable annual crops, and the incorporation of residues from cultivated plants such as alfalfa and faba bean. Comparable findings were reported by Buerkert et al. [60] and Luedeling et al. [61], who documented increased OM levels in oasis soils in Oman, attributing this to long-term annual manure applications. Compared to soils from modern oasis (S2, S6, S7, S9 and S12), the higher total soil nitrogen (N) content, particularly in S3, S4 and S5 soils may be explained by greater overall vegetation biomass, leading to higher carbon and nitrogen inputs into the soil. In contrast, soils from modern oasis exhibited lower OM and total N content, likely due to minimal additions of organic amendments, which may contribute to a long-term decline in soil fertility.

*Vicia faba* plants were grown in twelve soil samples from different oasis ecosystems, and the results indicated that S4 and S5 soils were the most suitable for plant growth, yielding the highest crop production. In these sites, *Vicia faba* has traditionally been cultivated, suggesting that the observed results may be attributed to the abundance and effectiveness of indigenous rhizobia. Owing to their symbiotic potentialities and ability to grow over a broad range of climatic and soil conditions, faba bean is extensively used in various cropping system such as intercropping or crop rotation to improve soil fertility and reduce the consumption of commercial N fertilizer [62,63]. These crops are well nodulated in several Tunisian soils; however, this is not always the case for oasis soils. No studies have previously investigated *Vicia faba* nodule-associated bacteria in oasis. Therefore, special attention has been given to this topic, particularly to the effectiveness of nodulating rhizobia in these environments.

In this study, 60 strains were isolated from faba bean root-nodules growing in 12 oasis soils. The phylogenetic identification highlighted a predominance of bacteria belonging to the genera *Pseudomonas* and *Bacillus*, which have been frequently isolated from root-nodules in various legumes including grass pea, lupine, alfalfa, soybean [64–66] as well as the Saharan leguminous tree *Vachelliatortilis* sub sp. *Raddiana* [6]. It has been reported that *Pseudomonas* spp. are among the most abundant members of bacterial communities associated with the date palm rhizosphere soil in Tunisian oasis [67]. *Pseudomonas* genera were observed at all sampling sites followed by *Bacillus*, which was reported in 11 out of 12 oasis, suggesting the adaptation of these genera to the oasis’s environment. On the other hand, *Hyphomicrobiales*, *Xanthomonadales*, *Burkholderiales* and *Rhodospirillales* orders have all been observed to be associated with the root-nodules of *V. faba* plants, suggesting that soil is the main microbial reservoir. In fact, in arid ecosystems geoclimatic conditions and agricultural management practices are of major importance in shaping the diversity and functionality of plant-associated bacterial communities [68]. Several studies have indicated that, *Pantoea*, *Bacillus, Pseudomonas, Agrobacterium*, *Serratia*, *Stenotrophomonas* spp. and many other bacteria live inside legume nodules [62,69]. This study also suggested a wide diversity of bacterial communities that interact in association with faba bean.

The PGP properties of bacteria have been studied in order to select strains with high potential to be used as biofertilizers. In our study, in vitro PGP characterization showed that all the strains had the ability to produce IAA ranged from 0.98 to 70.71 μg ml^−1^ and the highest producer of IAA was the strain affiliated to *K. radicincitans*. Results are similar to those obtained by Abdelkrim et al. [70] and Ferchichi et al. [65] who observed values from 0.89 to 63.55 μg ml^−1^and 0.67 to 74.51 μg ml^-1^, respectively, in bacteria isolated from grass pea and lupine root nodules, suggesting a potential symbiosis and closer relationship between bacterial strains and their hosts. In this study, all the strains isolated from *Vicia faba* root nodules were capable of producing IAA. This is in agreement with the observations of Saidi et al. [62] on faba bean root-nodules. It is estimated that up to 80% of bacteria isolated from the rhizosphere were able to synthesize IAA, a key plant hormone [71].

Siderophore production was the second most common plant growth-promoting trait observed, often co-occurring with auxin production, as reported in previous studies [70,72]. Additionally, 25% of bacterial strains produced hydrogen cyanide (HCN), a key biocontrol metabolite against fungal pathogens [16]. Among the identified genera, *Pseudomonas* exhibited the highest plant growth-promoting potential [65,73,74]. One-quarter of isolates displayed cellulase activity, facilitating root colonization, though bacterial entry is often linked to natural root cracks or root hairs [75,76]. The highest cellulase activity was detected in *Bacillus* strains, which also produced exopolysaccharides linked to stress tolerance [6,77,78]. Lipase production was detected in 43% of isolates, with *B. halotolerans* strains showing optimal activity [79,80]. Ammonia production, contributing to plant nitrogen supply, was highest in *G. frateurii* Vf47 and some *B. halotolerans* strains, which also exhibited strong phosphate-solubilizing activity [81,82].

Among the bacteria promoting plant growth, *K. radicincitans* was the least common genus, isolated only from root nodules of plants grown in S11 soil. Notably, this strain exhibits multiple plant growth-promoting traits. Indeed, several *Kosakonia* members are diazotrophs and promiscious endophytes that exhibit various PGP properties [83–86], which may promote plant growth directly or indirectly or synergistically [87]. Recently, the genus *Kosakonia* gained attention as the analysis of a few recently available genomes has shown interesting features that would support their PGP properties [84,88].

In this study, we identified four bacterial strains capable of re-nodulating and fulfilling the nitrogen requirements of their original host. These strains were closely related to *Rhizobium leguminosarum* and *Rhizobium laguerreae*, suggesting that they may be the primary nitrogen-fixing symbionts of faba bean in Tunisian oasis soils. These findings align with those of Belhadi et al. [89]. However, these strains accounted for only 8% of the total isolates, highlighting the high prevalence of non-rhizobial species in legume root nodules. Importantly, *R. laguerreae* showed high symbiotic efficiency on *Vicia faba* plants and promoted a significant increase in growth parameters. Similarly, previous reports have shown that faba bean was able to form an efficient nitrogen fixing symbiosis with *R. laguerreae* in Greece, Algeria, Tunisia, Peru and Spain [89–91]. This species was proposed for legumes-nodulating strains for *Pisum sativum* [72], *Lens culinaris* [92] *Phaseolus vulgaris* [93]. In addition to its symbiotic nitrogen fixation efficiency with legume partners, *R. laguerreae* showed also notable *in vitro* PGP activities such as the ability to produce IAA, siderophores, ammonia, ACC deaminase and exopolysaccharides. Results also revealed that plants inoculated with *K. radicincitans*, *Bacillus halotolerans* and *Pseudomonas fluorescens* showed a significant increase in both shoot and root dry biomass that could be due to their various PGP properties. In fact, *K. radicincitans* has been extensive discovery in many crops of global economic relevance most notably sugarcane, cotton, maize, rice, wheat and sweet potato, with the ability to interact and elicit beneficial effects on plant growth [94]. Similarly, *P. fluorescens* strains are well-known for their plant growth promotion and biocontrol potential, and are already included in many microbial bioinoculant products.

In the current study, some bacterial combinations promoted one or more growth parameters of *Vicia faba* when grown in two contrasting oasis soils with varying fertility levels. The positive effect of combined bacterial treatments on plant biomass, nodulation and nitrogen fixation is becoming a current trend. A meta-analysis by Kaschuk et al. [95] highlighted the role of various *Bacillus* strains in enhancing rhizobial symbiosis worldwide. It confirmed that co-inoculation of rhizobia and *Bacillus* species is a common strategy to boost plant growth, biological nitrogen fixation, nutrient acquisition, and ultimately increase grain legume yields. Herein, improved root growth with higher nodulation noted in plants inoculated with *R. laguerreae*+ *B*. *halotolerans* may have been consequence of increased IAA production, which stimulates plant cell division and elongation stimulating plant root elongation that results in enhancing mineral and nutrient uptake thus greatly contributes to the plant growth. Several studies have indicated that *Bacillus* strains produce IAA and other plant growth-promoting properties. Results are in agreement with findings of Taha et al. [15] who demonstrated that the combination of *R. laguerreae* with *Bacillus* sp and *E. aerogenes* increased plants biomass and the number of nodules.

Known for its multifaceted effects, bacterial IAA also plays a critical role in enhancing nodule formation, increasing rhizobial competitiveness for nodulation, prolonging the functional lifespan of nodules by delaying senescence, and inducing the expression of genes associated with legume–rhizobia symbiosis, thereby contributing significantly to efficient biological nitrogen fixation [96]. It also promotes root elongation and increases infection sites prior to nodulation while functioning as a signalling molecule in plant–bacteria interactions [97]. In the present study, improved overall plant growth, nodule dry wight and nitrogen accumulation were observed, likely due to elevated IAA production, which supports nodule development and function, [98]. According to Subramanian et al. [99], high levels of IAA produced by *B. megaterium* can be considered to have aided in the development of mature nodules, which thereby improved the nodular nitrogen fixation.

Biofertilizers offer considerable potential to enhance global food production; however, their large-scale adoption remains constrained by inconsistent field performance and economic limitations. The variability in microbial inoculant efficacy under fluctuating environmental conditions underscores the need to transition from controlled laboratory studies to extensive field-based validation trials [100]. Advances in formulation technologies are critical for improving microbial viability, stability, and shelf life, while the selection of suitable carriers and cost-effective production strategies is essential for scalability and accessibility, particularly for smallholder farmers [101]. Interdisciplinary collaboration between academia and industry, integrating expertise from engineering, materials science, biology, and agronomy, is crucial for developing robust and adaptable bioformulations [102]. A key challenge for the scientific community and industry is the development of tools and technologies that enable farmers to effectively utilize these microorganisms. Furthermore, enhancing farmer awareness through training programs and extension services can disseminate best practices for inoculant use emphasizing their long-term benefits for soil health and sustainability [99].

## Conclusions

Sixty bacterial strains were isolated from root-nodules of *Vicia faba* L. var. *minor* Saber 02 plants grown in 12 oasis soils. The 16S rDNA sequencing revealed seven bacterial orders: *Enterobacteriales, Pseudomonodales*, *Burkholderiales*, *Xanthomonadales*, *Rhodospirillales*, *Hyphomicrobiales*, *Bacillales*.

Particularly, five strains belonging to *R. laguerreae* (Vf19), *B. halotolerans* (Vf43), *G. frateurii* (Vf47), *P. reinekei* (Vf48) and *K. radicincitans* (Vf49) exhibited multiple PGP traits, interesting biocontrol potential as well as high tolerance to salt and drought stresses.

Eleven inoculums, consisting of efficient and salt tolerant PGPR combinations for inoculating *Vicia faba* L. var. *minor* Saber 02 in soils with contrasting fertility, demonstrated that co-inoculation with *R. laguerreae* Vf19 and *B. halotolerans* Vf43 significantly increased shoot biomass and nitrogen content. Additionally, inoculation with consortia of *R. laguerreae* Vf19 + *B. halotolerans* Vf43, and *R. laguerreae* Vf19 + *B. halotolerans* Vf43 + *K. radicincitans* Vf49, enhanced total soil nitrogen, particularly in low-fertility soils.

These findings highlight the potential of the PGPR–*V. faba* symbiosis as a promising candidate for developing effective biofertilizers tailored to oasis ecosystems and arid environments. Such bio inoculation strategies could be incorporated into oasis soil management programs, promoting sustainable agriculture by enhancing growth performance, and soil health, particularly in low-fertility oasis soils.

Current research is directed toward evaluating the potential of the selected bacterial strains, applied individually or in consortia, in enhancing *Vicia faba* growth performance, yield parameters as well as soil health under field conditions in arid regions. These studies also aim to elucidate their impact on the composition and functional dynamics of the indigenous soil microbiota.

## Supporting information

S1 Table Characteristics of bacterial strains isolated from *Vicia faba* root nodules. (DOCX)

S2 Table Tolerance to acid and alcaline pH, high temperature, salt and drought stress of bacteria isolated from *Vicia faba* root-nodules. (DOCX)
