# Supplementary material for: Characterization of plant growth-promoting bacteria from Vicia faba root nodules grown on oasis soils and their potential to enhance soil fertility and crop growth attributes in arid regions
Source: PLoS One. 2026 Jul 14;21(7):e0353365. doi: 10.1371/journal.pone.0353365 (PMC13367724; doi:10.1371/journal.pone.0353365)
Supplement: S1 Table — (DOCX) [file pone.0353365.s001.docx]

**S1 Table.** **Characteristics of bacterial strains isolated from *Vicia faba* root nodules.** IAA: Indoleacetic acid production, siderophore production, phosphate solubilization capacity, cellulase activity, ammonia production, HCN: Hydrogen cyanide production, N2: Nitrogen fixation, ACC: Deaminase activity, EPS, exopolysaccharide production, LA: Lipolytic activity and genus based on partial 16S rRNA gene sequence for each strain

| **Strain** | **IAA** | **Siderophore** | **Phosphate solubilization** | **Cellulase** | **Ammonia** | **HCN** | **N2**  **fixation** | **ACC deaminase** | **EPS** | **LA** | **Genus/ species**  **affiliation** | **% of similarity** | **GenBank**  **Accession number** |
| --- | --- | --- | --- | --- | --- | --- | --- | --- | --- | --- | --- | --- | --- |
| *Vf*1 | 53.18c | 12.27lm | nd | - | + | - | ++ | ++ | +++ | +++ | *Stenotrophomonas maltophilia* | 99.91% | ON411688 |
| *Vf*2 | 6.57kl | 12.5lm | nd | - | ++ | + | + | + | ++ | ++ | *Pseudomonas baetica* | 99.72% | ON411689 |
| *Vf*3 | 14.18i | 16.34k | nd | + | + | +++ | + | + | + | ++ | *Pseudomonas baetica* | 99.72 % | ON411690 |
| *Vf*4 | 12.20ij | 10.40mn | nd | + | - | + | ++ | + | +++ | - | *Rhizobium leguminosarum* | 99.90 % | ON411691 |
| *Vf*5 | 3.27lm | 16.37k | nd | - | - | - | - | + | ++ | - | *Pantoea agglomerans* | 99.62 % | ON411692 |
| *Vf*6 | 18.56h | 20.47ij | 4.67g | - | - | - | - | + | ++ | - | *Pantoea agglomerans* | 100 % | ON411693 |
| *Vf*7 | 9.24jk | 12.70lm | nd | - | + | +++ | - | - | + | + | *Pseudomonas koreensis* | 99.81 % | ON411694 |
| *Vf*8 | 2.21m | 16.11k | nd | - | + | - | - | - | + | +++ | *Pseudomonas moraviensis* | 99.62 % | ON411695 |
| *Vf*10 | 1.90m | nd | nd | - | + | - | +++ | ++ | +++ | - | *Pseudomonas moraviensis* | 99.81 % | ON411696 |
| *Vf*11 | 6.66kl | 12.5lm | 5.84f | - | - | - | ++ | + | + | - | *Raoultella terrigena* | 99.53 % | ON411697 |
| *Vf*12 | 6.12kl | 8.67n | 1.94i | ++ | ++ | - | + | - | + | - | *Pseudomonas putida* | 99.62 % | ON411698 |
| *Vf*13 | 25.08g | 25.84ef | nd | + | ++ | +++ | ++ | ++ | ++ | - | *Pseudomonas fluorescens* | 100 % | ON411699 |
| *Vf*14 | 6.86kl | 10.50lmn | nd | + | ++ | + | +++ | + | +++ | - | *Rhizobium leguminosarum* | 99.81 % | ON411700 |
| *Vf*15 | 18.30h | 8.07n | nd | - | - | - | +++ | ++ | +++ | - | *Rhizobium pusense* | 100% | ON411701 |
| *Vf*16 | 31.52ef | 34.67c | 1.94i | + | + | - | +++ | ++ | ++ | - | *Ensifer adhaerens* | 100 % | ON411702 |
| *Vf*17 | 6.05kl | 16,30k | nd | - | + | + | +++ | ++ | +++ | - | *Rhizobium pusense* | 100 % | ON411703 |
| *Vf*18 | 13.13i | 11.57lm | nd | - | + | +++ | + | - | ++ | - | *Pseudomonas baetica* | 99.81 % | ON411704 |
| *Vf*19 | 25.36g | 23.51fgh | nd | + | ++ | + | +++ | +++ | +++ | - | *Rhizobium laguerreae* | 99. 90 % | ON411705 |
| *Vf20* | 3.66lm | 4.87o | nd | - | +++ | - | ++ | ++ | +++ | - | *Rhizobium pusense* | 100 % | ON411706 |
| *Vf2*1 | 13.17i | 13.10l | nd | - | ++ | +++ | + | - | + | ++ | *Pseudomonas koreensis* | 99.81 % | ON411707 |
| *Vf*22 | 6.88kl | 3.64o | nd | + | ++ | +++ | + | + | ++ | - | *Pseudomonas koreensis* | 99.81 % | ON411708 |
| *Vf2*3 | 34.59ef | 44.84b | nd | + | ++ | ++ | ++ | ++ | +++ | - | *Pseudomonas koreensis* | 99.53 % | ON411709 |
| *Vf2*4 | 12.87ij | 25.07efgh | nd | - | + | - | +++ | ++ | +++ | - | *Rhizobium laguerreae* | 99.81 % | ON411710 |
| *Vf2*5 | 15.11hi | 16.57k | nd | - | + | +++ | - | - | + | ++ | *Pseudomonas reinekei* | 99.62 % | ON411711 |
| *Vf2*6 | 0.98m | 16.40k | nd | - | + | + | - | - | - | + | *Xanthomonas translucens* | 100 % | ON411712 |
| *Vf2*8 | 31.50f | nd | nd | - | +++ | + | + | + | + | - | *Rhizobium pusense* | 100 % | ON411713 |
| *Vf2*9 | 39.26d | 16.67k | nd | - | + | - | ++ | ++ | +++ | +++ | *Stenotrophomonas maltophilia* | 100 % | ON411714 |
| *Vf*30 | 24.38g | 26.01ef | nd | - | + | - | ++ | + | +++ | - | *Achromobacter xylosoxidans* | 99.91 % | ON411715 |
| *Vf3*1 | 6.60kl | 26.39e | 7.17e | + | + | - | ++ | + | +++ | + | *Klebsiella pneumoniae* | 99.81 % | ON411716 |
| *Vf3*2 | 33.62ef | 22.37hi | nd | - | + | - | +++ | ++ | +++ | + | *Achromobacter xylosoxidans* | 100 % | ON411717 |
| *Vf3*3 | 3.54lm | 17.69k | 10.11c | - | + | - | ++ | + | +++ | - | *Klebsiella pneumoniae* | 99.81 % | ON411718 |
| *Vf3*4 | 35.29e | 22.34hi | nd | - | - | - | ++ | +++ | +++ | - | *Achromobacter xylosoxidans* | 100 % | ON411719 |
| *Vf3*5 | 1.19m | 11.83lm | 8.10d | +++ | +++ | - | + | - | + | +++ | *Bacillus halotolerans* | 100 % | ON411720 |
| *Vf3*6 | 1.06m | 12.4lm | nd | - | + | - | - | - | + | +++ | *Pseudomonas aeruginosa* | 100 % | ON411721 |
| *Vf3*7 | 7.02kl | 25.31efg | nd | ++ | - | - | + | - | ++ | - | *Bacillus halotolerans* | 100 % | ON411722 |
| *Vf3*8 | 25.18g | 22.40hi | 17.67b | - | ++ | - | - | - | - | - | *Gluconobacter frateurii* | 100 % | ON411723 |
| *Vf3*9 | 12.66ij | 12.00lm | nd | - | - | - | + | + | - | - | *Serratia marcescens* | 99.91 % | ON411724 |
| *Vf*40 | 6.35kl | 45.35b | nd | - | - | - | + | - | ++ | - | *Brucella intermedia* | 100 % | ON411725 |
| *Vf4*1 | 6.78kl | 18.60k | nd | - | ++ | - | +++ | ++ | +++ | - | *Rhizobium pusense* | 100 % | ON411726 |
| *Vf4*2 | 40.37d | 26.50e | nd | - | + | - | +++ | ++ | +++ | - | *Rhizobium pusense* | 100 % | ON411727 |
| *Vf4*3 | 39.62d | 31.00d | nd | +++ | + | - | ++ | + | - | +++ | *Bacillus halotolerans* | 100 % | ON411728 |
| *Vf*44 | 13.76i | 12.00lm | nd | + | ++ | - | ++ | + | + | ++ | *Bacillus subtilis* | 99.62 % | ON411729 |
| *Vf4*5 | 3.33lm | 48.84a | nd | ++ | - | - | + | + | ++ | +++ | *Bacillus halotolerans* | 99.44 % | ON411730 |
| *Vf4*6 | 3.44lm | 47.50ab | nd | - | ++ | - | + | ++ | ++ | - | *Pseudomonas aeruginosa* | 99.62 % | ON411731 |
| *Vf4*7 | 12.73ij | 12.00lm | 25.01a | - | +++ | ++ | ++ | + | + | - | *Gluconobacter frateurii .* | 100 % | ON411732 |
| *Vf4*8 | 13.86i | 49.00a | nd | - | +++ | +++ | + | - | + | - | *Pseudomonas reinekei* | 99.62 % | ON411733 |
| *Vf4*9 | 70.71a | 35.00c | 8.17d | - | - | - | +++ | +++ | ++ | - | *Kosakonia radicincitans* | 100 % | ON411734 |
| *Vf5*1 | 6.69kl | 17.66jk | nd | +++ | ++ | + | + | ++ | ++ | ++ | *Bacillus halotolerans* | 100 % | ON411735 |
| *Vf5*2 | 31.14f | 22.39hi | 3.20h | +++ | +++ | + | ++ | ++ | + | +++ | *Bacillus halotolerans* | 99.72 % | ON411736 |
| *Vf5*3 | 6.63kl | 16.67k | nd | +++ | +++ | + | - | - | + | ++ | *Bacillus tequilensis* | 99.91 % | ON411737 |
| *Vf5*4 | 14.25i | 44.83b | 2.94h | +++ | +++ | + | ++ | - | +++ | +++ | *Bacillus halotolerans* | 100 % | ON411738 |
| *Vf*55 | 14.19i | 25.47ef | 10.60c | - | - | ++ | - | - | + | + | *Serratia marcescens* | 99.81 % | ON411739 |
| *Vf5*6 | 6.69kl | 16.40k | nd | + | + | - | + | - | ++ | + | *Bacillus halotolerans* | 100 % | ON411740 |
| *Vf5*7 | 6.55kl | 18,35jk | nd | ++ | ++ | - | - | - | ++ | ++ | *Bacillus subtilis* | 99.91 % | ON411741 |
| *Vf5*9 | 6.79kl | 22.70ghi | nd | ++ | ++ | - | + | + | + | ++ | *Bacillus safensis* | 100 % | ON411742 |
| *Vf*60 | 14.12i | 12.00lm | 1.37i | - | ++ | - | + | + | - | - | *Pseudomonas baetica* | 99.44 % | ON411743 |
| *Vf*62 | 62.97b | 12.40lm | nd | - | ++ | - | ++ | - | + | - | *Brucella haematophila* | 100 % | ON411744 |
| *Vf*63 | 6.85kl | 11.73lm | nd | + | - | - | ++ | + | + | +++ | *Stenotrophomonas maltophilia* | 99.63 % | ON411745 |
| *Vf*64 | 2.18m | 12.17lm | nd | - | - | - | - | - | - | - | *Bacillus subtilis* | 100 % | ON411746 |
| *Vf*65 | 18.16h | 10.27mn | nd | + | +++ | - | + | - | - | - | *Bacillus halotolerans* | 100 % | ON411747 |

Values are means of three replicates. Intensity of production indicator: none, −; weak, +; moderate, ++; strong, +++. Means followed by a common lowercase letter are not significantly different at *p* < 0.05, according to Tukey’s HSD test,. nd, not detected activities.
