# Supplementary material for: Characterization of plant growth-promoting bacteria from Vicia faba root nodules grown on oasis soils and their potential to enhance soil fertility and crop growth attributes in arid regions
Source: PLoS One. 2026 Jul 14;21(7):e0353365. doi: 10.1371/journal.pone.0353365 (PMC13367724; doi:10.1371/journal.pone.0353365)
Supplement: S2 Table — (DOCX) [file pone.0353365.s002.docx]

**S2 Table.** **Tolerance to acid and alcaline pH, high temperature,** **salt and drought stress of bacteria isolated from *Vicia faba* root-nodules.**

| **Strain** | Max NaCl (mM) | pH min | PH max | T° max | PEG 30% | **Strain** | Max NaCl (mM) | pH min | PH max | T° max | PEG 30% |
| --- | --- | --- | --- | --- | --- | --- | --- | --- | --- | --- | --- |
| *Vf*1 | 800 | 5 | 10 | 40 | - | *Vf3*3 | 700 | 5 | 10 | 45 | + |
| *Vf*2 | 1000 | 4 | 10 | 45 | +++ | *Vf3*4 | 1000 | 4 | 10 | 45 | - |
| *Vf*3 | 1000 | 4 | 10 | 45 | +++ | *Vf3*5 | 1000 | 4 | 10 | 45 | + |
| *Vf*4 | 900 | 5 | 10 | 45 | ++ | *Vf3*6 | 1000 | 5 | 10 | 45 | +++ |
| *Vf*5 | 700 | 5 | 10 | 45 | ++ | *Vf3*7 | 1000 | 5 | 10 | 45 | +++ |
| *Vf*6 | 800 | 4 | 10 | 45 | ++ | *Vf3*8 | 900 | 4 | 10 | 45 | + |
| *Vf*7 | 1000 | 4 | 10 | 45 | +++ | *Vf3*9 | 1000 | 4 | 10 | 45 | - |
| *Vf*8 | 1000 | 4 | 10 | 45 | ++ | *Vf*40 | 900 | 4 | 10 | 45 | ++ |
| *Vf*10 | 1000 | 4 | 10 | 45 | +++ | *Vf4*1 | 1000 | 4 | 10 | 45 | +++ |
| *Vf*11 | 800 | 5 | 10 | 45 | + | *Vf4*2 | 1000 | 4 | 10 | 45 | +++ |
| *Vf*12 | 1000 | 4 | 10 | 45 | ++ | *Vf4*3 | 1000 | 5 | 10 | 45 | +++ |
| *Vf*13 | 1000 | 4 | 10 | 45 | +++ | *Vf*44 | 1000 | 6 | 10 | 45 | +++ |
| *Vf*14 | 900 | 5 | 10 | 45 | ++ | *Vf4*5 | 1000 | 4 | 10 | 45 | +++ |
| *Vf*15 | 1000 | 4 | 10 | 45 | +++ | *Vf4*6 | 1000 | 5 | 10 | 45 | +++ |
| *Vf*16 | 800 | 5 | 10 | 45 | ++ | *Vf4*7 | 900 | 4 | 10 | 45 | + |
| *Vf*17 | 1000 | 4 | 10 | 45 | +++ | *Vf4*8 | 1000 | 4 | 10 | 45 | +++ |
| *Vf*18 | 1000 | 4 | 10 | 45 | +++ | *Vf4*9 | 1000 | 4 | 10 | 45 | ++ |
| *Vf*19 | 900 | 5 | 10 | 45 | ++ | *Vf5*1 | 1000 | 5 | 10 | 45 | +++ |
| *Vf20* | 1000 | 5 | 10 | 45 | ++ | *Vf5*2 | 1000 | 5 | 10 | 45 | +++ |
| *Vf2*1 | 1000 | 5 | 10 | 45 | +++ | *Vf5*3 | 1000 | 5 | 10 | 45 | +++ |
| *Vf*22 | 1000 | 5 | 10 | 45 | +++ | *Vf5*4 | 1000 | 5 | 10 | 45 | +++ |
| *Vf2*3 | 1000 | 5 | 10 | 45 | +++ | *Vf*55 | 1000 | 5 | 10 | 45 | ++ |
| *Vf2*4 | 900 | 4 | 10 | 45 | ++ | *Vf5*6 | 1000 | 5 | 10 | 45 | ++ |
| *Vf2*5 | 1000 | 4 | 10 | 45 | ++ | *Vf5*7 | 1000 | 5 | 10 | 45 | ++ |
| *Vf2*6 | 700 | 5 | 10 | 40 | - | *Vf5*9 | 1000 | 6 | 10 | 45 | +++ |
| *Vf2*8 | 1000 | 4 | 10 | 45 | +++ | *Vf*60 | 1000 | 4 | 10 | 45 | +++ |
| *Vf2*9 | 900 | 4 | 10 | 40 | - | *Vf*62 | 1000 | 4 | 10 | 45 | ++ |
| *Vf*30 | 1000 | 4 | 10 | 45 | + | *Vf*63 | 800 | 5 | 10 | 40 | - |
| *Vf3*1 | 800 | 4 | 10 | 45 | + | *Vf*64 | 1000 | 5 | 10 | 45 | +++ |
| *Vf3*2  Intensity of tolerance indicator: none, −; weak, +; moderate, ++; strong, +++ | 1000 | 4 | 10 | 45 | + | *Vf*65 | 1000 | 5 | 10 | 45 | ++ |
